# Supplementary material for: Comparative analysis of profitability and resource use efficiency between Penaeus monodon and Litopenaeus vannamei in India
Source: PLoS One. 2021 May 4;16(5):e0250727. doi: 10.1371/journal.pone.0250727 (PMC8096089; doi:10.1371/journal.pone.0250727)
Supplement: S2 File — (DOCX) [file pone.0250727.s002.docx]

**Ocean University of China**

No. 5 Yushan Road, Qingdao 266 003, China

**Production and Value chain Analysis of Farmed Shrimp in Gujarat (India)**

I. Socioeconomic Profile of Sample farmers

1. Personal Information of Head of Household/respondent
2. Name:_______________________________________________Age:___
3. Address:_____________________________________________________
4. Contact No.:__________________________________________________
5. Occupation:___________________________________________________
6. Education: Illiterate Primary Secondary Higher Secondary Graduate
7. Family Specification
8. Family Type: Joint Nuclear
9. Members

Adult: Male____________Female_____________

Children(Less than 14 yrs.): Male____________Female_____________

1. Occupations of family members

| Sl. No. | Members | Govt. Job | Business | Private Job | Agriculture | Fisheries |
| --- | --- | --- | --- | --- | --- | --- |
|  | Male |  |  |  |  |  |
|  | Female |  |  |  |  |  |

**II: Land inventory** (in ha.) (1 Acre=________ha.)

1. Total Land Holdings: Owned:_______________Leased in:___________

Leased out:________Lease rent_____________ Leasing period______________

1. Total area under **pond**: Owned:_______________Leased in:___________

Leased out:________Lease rent_____________ Leasing period______________

1. Area under **Shrimp Farming**:Owned:________Leased in:_______
2. Area under (a) L.: **vannamei Farming**: ________ (b) P. monodon::______

Questionnaire for shrimp farmers in Gujarat

1. Are you farming under

Intensive mode Semi-Intensive mode Extensive mode

1. What is the culture duration?
2. When does it begin and end?
3. From where do you buy your seeds? Name:_------------------------ Distance
4. Why do choose those seed suppliers?.................................................

- For lower price □ Higher quality
- Near your farm □ Other reasons

1. To whom do you sell your shrimp?

- Processor □ Local market
- Middle men □ Others (______________)

1. ***.Fixed investment on Farm/Farm inventory:***

| Particulars | No. | Year of construction/purchase | Cost of purchase or construction /PV(Rs.) | Expected life (Yr) | Annual repair/ maintenance Cost (Rs) |
| --- | --- | --- | --- | --- | --- |
| Ponds construction |  |  |  |  |  |
| Reservoir |  |  |  |  |  |
| Effluent treatment system |  |  |  |  |  |
| Bio-security measures |  |  |  |  |  |
| Inlet/outlet |  |  |  |  |  |
| Farm building |  |  |  |  |  |
| Power connection |  |  |  |  |  |
| Lightening of farm |  |  |  |  |  |
| Generator set |  |  |  |  |  |
| Bore well |  |  |  |  |  |
| Pump set/tube well |  |  |  |  |  |
| Aerator |  |  |  |  |  |
| Feeding Boat |  |  |  |  |  |
| Mechanical feeder |  |  |  |  |  |
| Nets |  |  |  |  |  |
| Minor implements |  |  |  |  |  |
| Transport vehicle |  |  |  |  |  |
| Tractor |  |  |  |  |  |
|  |  |  |  |  |  |

**B. Sources of capital**: Own/credit

(a) Loan from friends :-------------(b) Loan from money lender: Rs. (c) Loan Bank: Rs.

| **Purpose** | **Loan amount (Rs. thousand)** | **Source** | **Repayment period (yrs)** | **Rate of interest** |
| --- | --- | --- | --- | --- |
|  |  |  |  |  |
|  |  |  |  |  |

1. **Stocking and Sources of seed**

| Date of stocking | Area stocked | *Seed* | | Price (Rs./piece) | Distance of Source of seed (Km) | Mode of transport | Transportn cost (Rs) |
| --- | --- | --- | --- | --- | --- | --- | --- |
|  |  | *Stage* | No./m*^2^* |  |  |  |  |
|  |  |  |  |  |  |  |  |
|  |  |  |  |  |  |  |  |
|  |  |  |  |  |  |  |  |
|  |  |  |  |  |  |  |  |

1. **Feeding Practices**

| Feed | Method of feeding | Frequency of feeding  (No./day) | FCR | Total quantity of feed used  (q) | Price (Rs./kg) | Distance of feed Source | Transportn. cost (Rs) |
| --- | --- | --- | --- | --- | --- | --- | --- |
|  |  |  |  |  |  |  |  |
|  |  |  |  |  |  |  |  |
|  |  |  |  |  |  |  |  |
|  |  |  |  |  |  |  |  |

1. **Details of inputs used in one full cycle of crop**

| Particulars | Method of application | No. of times applied  (No.) | Quantity applied each time  (Kg/time) | Total quantity applied  (Kg) | Price including transport  (Rs./Kg) |
| --- | --- | --- | --- | --- | --- |
| Bleaching powder |  |  |  |  |  |
| Lime (kg) |  |  |  |  |  |
| Zypsum (CaSO4) (kg) |  |  |  |  |  |
| Dolomite |  |  |  |  |  |
| Magnesium |  |  |  |  |  |
| Potassium (MOP) |  |  |  |  |  |
| Potassium permagnet (PP) |  |  |  |  |  |
| Feed Probiotics |  |  |  |  |  |
| SoilProbiotics |  |  |  |  |  |
| Water Probiotics |  |  |  |  |  |
| Medicines |  |  |  |  |  |
|  |  |  |  |  |  |
|  |  |  |  |  |  |
|  |  |  |  |  |  |

1. **Details of Power and human labour used in one full crop cycle**

| Particulars | Details | Total quantity used/crop | Price (Rs./unit) |
| --- | --- | --- | --- |
| Electricity charges |  |  |  |
| Fuel charge | 1.Diesel (litre) |  |  |
| Permanent labour | Technical staff |  |  |
|  | Skilled labour |  |  |
| Casual/ contractual Labour | Hired labour : Male  Female |  |  |
| Communication expense (Rs.) | |  |  |

**G. Harvesting of shrimp**

| Date of | | Crop duration (days) | Area of Harvest (Acre) | Production | | Sold at farm /processing plant | Agency to whom sold | Harvesting charges |
| --- | --- | --- | --- | --- | --- | --- | --- | --- |
| Harvest | Seed stocking |  |  | Grade (count) | Quantity |  |  |  |
|  |  |  |  |  |  |  |  |  |
|  |  |  |  |  |  |  |  |  |
|  |  |  |  |  |  |  |  |  |
| Total |  |  |  |  |  |  |  |  |

**H.Disposal pattern of last year production**

| Particulars | *P. monodon* | *L. vennemai* |
| --- | --- | --- |
| Total production |  |  |
| Home consumption |  |  |
| Friends & others(Gift) |  |  |
| Wastage |  |  |
| Any other |  |  |
| Sold quantity |  |  |
| Price (Rs./Kg) |  |  |

**Harvesting Details:** Daily/weekly/monthly:

**Selling of last crop produce**

| Sl. No. | Production  (Qty) | Market | | | | | | Farm | | | | | |
| --- | --- | --- | --- | --- | --- | --- | --- | --- | --- | --- | --- | --- | --- |
|  |  | Wholesale | | auctioneer | | middleman | | Processor | | Agent | | Consumer | |
|  |  | Q | P | Q | P | Q | P | Q | P | Q | P | Q | P |
| 1. |  |  |  |  |  |  |  |  |  |  |  |  |  |
| 2. |  |  |  |  |  |  |  |  |  |  |  |  |  |

1. How much (%) do you sell to each buyer?

- Processor □ Local market
- Middle men □ Others

1. What problem do you face while doing shrimp farming?
2. Have you applied for loan from bank?

- Yes □ No

1. Do you learn culturing techniques and disease prevention before starting your business?

- Yes □ No
  If yes, from where do you learn?

1. Do you have any support from processor?

- Yes □ No

1. Does local government/NGO offer any aid programs to farmers?

- Yes □ No
  If yes who are they?

1. Do you look for market information related to your business?

- Yes □ No

15. If yes, where can you find the information that you need?

□ Print media □ Electronic media □ other farmers □ other source

1. Do you have to comply with any regulations during your shrimp farming?

|  | 2009 | 2010 | 2011 |
| --- | --- | --- | --- |
| Harvest |  |  |  |
| Farm-gate price |  |  |  |

17. Why do you sell your harvest to middlemen?
