# Supplementary material for: Comparative analysis of profitability and resource use efficiency between Penaeus monodon and Litopenaeus vannamei in India
Source: PLoS One. 2021 May 4;16(5):e0250727. doi: 10.1371/journal.pone.0250727 (PMC8096089; doi:10.1371/journal.pone.0250727)

PLOS ONE

Comparative Analysis of Profitability and Resource Use Efficiency for Penaeus Monodon vs Litopenaeus Vannamei in India

--Manuscript Draft--

**Manuscript Number:**	PONE-D-20-36703
**Article Type:**	Research Article
**Full Title:**	Comparative Analysis of Profitability and Resource Use Efficiency for Penaeus Monodon vs Litopenaeus Vannamei in India
**Short Title:**	Profitability and resource use efficiency of shrimp aquaculture in India
**Corresponding Author:**	Yongtong Mu<br>Ocean University of China Fisheries College<br>Qingdao, CHINA
**Keywords:**	Shrimp aquaculture; Cost concepts; Cobb-Douglas production function; Resource use efficiency; allocative efficiency; India
**Abstract:**	The study aimed to highlight the profitability and production function analysis of Penaeus monodon and Litopenaeus vannamei in intensified shrimp farms in Gujarat (India). 220 shrimp farm households were used to identify (through principal component and cluster analyses) 8 clusters of management practices that reflected various scales of production intensity ranging from 0-2999 kg/ha/crop to 9000kg/ha/crop and above for both the species,. The Cobb-Douglas production function, which relates production output to several independent input variables, was used to determine productivity. The budgeting analysis for both the species showed that more intensively managed farms outperformed the less intensive farm. Empirical results show feed as most significant input for Penaeus monodon and for Litopenaeus vannamei seed and labor that affected production. Average net returns/ha/year for Penaeus monodon was $16313.13 and for Litopenaeus vannamei was $41640.99. Aquaculture exhibited decreasing returns to scale for both the species and estimates on resource use efficiency revealed that in Penaeus monodon the resources were economically utilized and in case of Litopenaeus vannamei the output was likely to increase if more of seed and less of labor would have been used. The major constraint for the shrimp farmers was the disease problem that can be solved by optimum stocking densities and proper feed management.
**Order of Authors:**	Ubair Nisar
	Yongtong Mu
	Hongzhi Zhang
	Mahida Navghan
	Yugui Zhu
**Opposed Reviewers:**	
**Additional Information:**	
**Question**	**Response**
**Financial Disclosure**<br><br>Enter a financial disclosure statement that describes the sources of funding for the work included in this submission. Review the submission guidelines for detailed requirements. View published research articles from *PLOS ONE* for specific examples.	Yongtong Mu, acknowledge the financial support of the Ministry of Agriculture and Rural Affairs of Peoples Republic of China (CARS-49).<br>NO - The funders had no role in study design, data collection and analysis, decision to publish, or preparation of the manuscript.

Powered by Editorial Manager® and ProduXion Manager® from Aries Systems Corporation

This statement is required for submission and **will appear in the published article** if the submission is accepted. Please make sure it is accurate.<br><br>**Unfunded studies**<br>Enter: *The author(s) received no specific funding for this work.*<br><br>**Funded studies**<br>Enter a statement with the following details:<br>• Initials of the authors who received each award<br>• Grant numbers awarded to each author<br>• The full name of each funder<br>• URL of each funder website<br>• Did the sponsors or funders play any role in the study design, data collection and analysis, decision to publish, or preparation of the manuscript?<br>• **NO** - Include this sentence at the end of your statement: *The funders had no role in study design, data collection and analysis, decision to publish, or preparation of the manuscript.*<br>• **YES** - Specify the role(s) played.<br><br>* typeset	
**Competing Interests**<br><br>Use the instructions below to enter a competing interest statement for this submission. On behalf of all authors, disclose any competing interests that could be perceived to bias this work—acknowledging all financial support and any other relevant financial or non-financial competing interests.<br><br>This statement **will appear in the published article** if the submission is accepted. Please make sure it is accurate. View published research articles from *PLOS ONE* for specific examples.	The authors have declared that no competing interests exist

Powered by Editorial Manager® and ProduXion Manager® from Aries Systems Corporation

**NO authors have competing interests**<br><br>Enter: *The authors have declared that no competing interests exist.*<br><br>**Authors with competing interests**<br><br>Enter competing interest details beginning with this statement:<br><br>*I have read the journal's policy and the authors of this manuscript have the following competing interests: [insert competing interests here]*<br><br>* typeset	
**Ethics Statement**<br><br>Enter an ethics statement for this submission. This statement is required if the study involved:<br><br>• Human participants<br>• Human specimens or tissue<br>• Vertebrate animals or cephalopods<br>• Vertebrate embryos or tissues<br>• Field research<br><br>Write "N/A" if the submission does not require an ethics statement.<br><br>General guidance is provided below. Consult the submission guidelines for detailed instructions. **Make sure that all information entered here is included in the Methods section of the manuscript.**	N/A

**Format for specific study types**<br><br>**Human Subject Research (involving human participants and/or tissue)**<br>• Give the name of the institutional review board or ethics committee that approved the study<br>• Include the approval number and/or a statement indicating approval of this research<br>• Indicate the form of consent obtained (written/oral) or the reason that consent was not obtained (e.g. the data were analyzed anonymously)<br><br>**Animal Research (involving vertebrate animals, embryos or tissues)**<br>• Provide the name of the Institutional Animal Care and Use Committee (IACUC) or other relevant ethics board that reviewed the study protocol, and indicate whether they approved this research or granted a formal waiver of ethical approval<br>• Include an approval number if one was obtained<br>• If the study involved *non-human primates*, add *additional details* about animal welfare and steps taken to ameliorate suffering<br>• If anesthesia, euthanasia, or any kind of animal sacrifice is part of the study, include briefly which substances and/or methods were applied<br><br>**Field Research**<br><br>Include the following details if this study involves the collection of plant, animal, or other materials from a natural setting:<br>• Field permit number<br>• Name of the institution or relevant body that granted permission	
**Data Availability**<br><br>Authors are required to make all data underlying the findings described fully available, without restriction, and from the time of publication. PLOS allows rare exceptions to address legal and ethical concerns. See the PLOS Data Policy and FAQ for detailed information.	No - some restrictions will apply

A Data Availability Statement describing where the data can be found is required at submission. Your answers to this question constitute the Data Availability Statement and **will be published in the article**, if accepted.<br><br>**Important:** Stating 'data available on request from the author' is not sufficient. If your data are only available upon request, select 'No' for the first question and explain your exceptional situation in the text box.<br><br>Do the authors confirm that all data underlying the findings described in their manuscript are fully available without restriction?	
**Describe where the data may be found in full sentences. If you are copying our sample text, replace any instances of XXX with the appropriate details.**<br><br>• If the data are **held or will be held in a public repository**, include URLs, accession numbers or DOIs. If this information will only be available after acceptance, indicate this by ticking the box below. For example: *All XXX files are available from the XXX database (accession number(s) XXX, XXX.*).<br>• If the data are all contained **within the manuscript and/or Supporting Information files**, enter the following: *All relevant data are within the manuscript and its Supporting Information files.*<br>• If neither of these applies but you are able to provide **details of access elsewhere**, with or without limitations, please do so. For example:<br><br>*Data cannot be shared publicly because of [XXX]. Data are available from the XXX Institutional Data Access / Ethics Committee (contact via XXX) for researchers who meet the criteria for access to confidential data.*<br><br>*The data underlying the results presented in the study are available from (include the name of the third party*	Data will be available on request

and contact information or URL).<br>• This text is appropriate if the data are owned by a third party and authors do not have permission to share the data.<br><br>* typeset	
Additional data availability information:	Tick here if your circumstances are not covered by the questions above and you need the journal’s help to make your data available.

Powered by Editorial Manager® and ProduXion Manager® from Aries Systems Corporation

中國海洋大學
OCEAN UNIVERSITY OF CHINA

COVERING LETTER

PLOS ONE

Dear Editor-in-Chief November 18, 2020

I would like to submit the manuscript entitled "**Comparative Analysis of Profitability and Resource Use Efficiency for *Penaeus Monodon* vs *Litopenaeus Vannamei* in India"** by author names to be considered for publication as an original **research article** in the PLOS ONE
The study highlights the comparative factor -product relationships in shrimp culture for both the species and this type of work has not been done before for India. Literature review suggests that earlier studies on shrimp aquaculture in the country did not gave deep insights of profitability, resource use efficiency and constraints, which have been presented in this study. The study gives a clear understanding of the shrimp farming and the factors associated with it and can help the existing farmers/ new entrepreneurs by making the venture more profitable. We believe these findings will be of interest to the readers of your journal.

We declare that this manuscript is original, has not been published before and is not currently being considered for publication elsewhere.

As Corresponding Author, I confirm that the manuscript has been read and approved for submission by all the named authors. We hope you find our manuscript suitable for publication and look forward to hearing from you in due course.

Corresponding Author: Dr. Yongtong Mu

Address: College of Fisheries, Faculty of Fisheries Economic and Management, Ocean University of China, Qingdao 266003, P.R. China

Tel No: +86-532-82032688,

E-mail: ytmu@ouc.edu.cn

Suggested reviewers:

1. **Kit Ming Isabel Yan**
   City University of Hong Kong
   HONG KONG

2. **Paolo Agnolucci**
   University College London
   UNITED KINGDOM

Manuscript Click here to access/download;Manuscript;Manuscript.docx

Comparative Analysis of Profitability and Resource Use Efficiency ~~for~~ *Penaeus Monodon* ~~vs~~ *Litopenaeus Vannamei* in India

Ubair Nisar [a] , Yongtong Mu [*a] , Hongzhi Zhang [b] , Mahida Navghan [c] , Yugui Zhu [a]

[a] *Key laboratory of Mariculture (Ministry of Education) College of Fisheries, Ocean University of China, No. 5 Yushan Road, Qingdao 266 003, China*

[b] *Shandong Foreign Trade Vocational College, Jufeng Road, Qingdao 266 100, China*

[c] *Central Institute of Fisheries Education, Versova, Mumbai- 400 061, India*

*Corresponding Author
Email: ytmu@ouc.edu.cn (YM)

Abstract

The study aimed to highlight the profitability and production function analysis of *Penaeus monodon* and *Litopenaeus vannamei* in intensified shrimp farms in Gujarat (India). ~~220~~ shrimp farm households were used to identify (~~through~~ principal component and cluster analyses) 8 clusters of management practices that reflected various scales of production intensity ranging from 0-2999 kg/ha/crop to 9000kg/ha/crop and above for both the species,. The Cobb-Douglas production function, which relates production output to several independent input variables, was used to determine productivity. The budgeting analysis for both the species showed that more intensively managed farms ~~outperformed~~ the less intensive farm. Empirical results show feed as most significant input for *Penaeus monodon* and ~~for~~ *Litopenaeus vannamei* seed and labor that affected production. Average net returns/ha/year for *Penaeus monodon* was $16313.13 and for *Litopenaeus vannamei* ~~was~~ $41640.99. Aquaculture exhibited decreasing returns to scale for both the species and estimates on resource use efficiency revealed that in *Penaeus monodon* the resources were economically utilized and in case of *Litopenaeus vannamei* the output was likely to increase if more of seed and less of labor would have been used. The major constraint for the shrimp farmers was ~~the disease problem that can be solved~~ by optimum stocking densities and proper feed management.

Keywords

Shrimp aquaculture, Cost concepts, Cobb-Douglas production function, Resource use efficiency, allocative efficiency, India

Introduction

The fisheries sector plays significant role in Indian economy contributing to 0.91% to national GDP and 5.23% to the agricultural GDP [1]. Indian fisheries and aquaculture is an important sector of food production that not only provides livelihood to around 14 million people but also contributes well in agricultural exports. Although the shrimp culture has increased during the past decade, the actual potential is still to be explored. The country is currently having 176,000 hectares of area under shrimp culture out of which around 91% is under the farming of *Litopenaeus vannamei*, 8% for *Penaeus monodon* and only 1% for *Macrobrachium Rosenberger* [2], Shrimp production can be increased by best utilizing the existing resources through improved practices of shrimp culture [3]. Shrimps are called the pinkish gold of the sea because of its increasing demand, great taste and high unit value realization in the export market. It is one of the immersing industry, which significantly contributes to foreign exchange and trade

Gujarat is the fourth largest shrimp producing state in India and emerged as one of the most productive and sustainable shrimp farming state [4]. The total crustaceans exported globally from India in the year 2019 was around 645 million tons worth of 4461 US million dollars (Fig 1). Over the last decade, there has been tremendous increase in the export of crustaceans majority of which constitutes of frozen seawater shrimps. It is observed in the Fig 1 that growth in the crustacean export has increased 4.5 times in terms of quantity and 5.3 times in terms of value since 2001. India also outpaced Indonesia, Thailand, and Ecuador to take the title for most shrimp exports to the U.S. for the fourth straight year [5]. It is a dire necessity of aquaculture to grow in order to provide food for growing population. However, the growth should be sustainable and hence responsible for the sustenance in the long run especially for the developing country like India. Studies explain the factors like production (output) intensities and farm sizes as the criterion for

the aquaculture sustainability and refer small-scale productions as low input or extensive while large scale production is referred as intensive [6,7]. The small-scale production systems mostly use household operated labor and do not rely on hired labors [8]. However, the low intensity farms can be converted into high efficiency production units and managed intensively as large sized by innovations and standardization of procedures [9]. It is argued [10, 11] that low intensity extensive farms tend to be more technically and economically efficient and fetch lower FCR ratio. Similar results were obtained by [12], revealing that though the cost of production per kilogram was highest in semi-intensive culture followed by intensive and extensive culture, the profit per kilogram was highest for extensive culture. Different results obtained by [13, 14, 15] revealed that extensive culture is much more profitable than semi-intensive and intensive culture. Shrimp production is mostly dependent on the stocking densities but does not solely influence the production levels [16, 17; 18]. High stocking densities and use of high inputs like pelletized feed and medicines characterize the intensive culture. Most of the farmers involved in the culture are urban entrepreneurs with elite businesses and large corporations. The farm owners do not personally take active participation in the management and instead hire managers and technical staff.

Fig 1. Performance of Indian crustacean export to the world (2001-201[illegible]

In the early 1980's due to high demand and prices of shrimps, the profit margins were very high which lured the investors towards this enterprise resembling a gold rush. However, this expanding industry started encountering the problems since 1988 [19]. Due to farm intensifications, the resource bases started degrading leading to disease outbreak which subsequently led to the drop of shrimp production. Thus, sustainability of shrimp production emerged as a prior concern for long-term viability of the business. The sustainability does not only include ecological sustainability

but economic sustainability also determining the capacity of the production system to produce a positive income for the long run.

This study was conducted to assess profitability and resource use efficiency of sample shrimp farms in Gujarat (India) and to determine performances across the level of intensification. ~~The specific objectives are to determine which inputs are the most important determinants of total output; the returns to scale and allocative efficiency of inputs used in shrimp aquaculture production.~~

~~Material and methods~~

Sampling procedure

The study was carried in the state of Gujarat possessing the largest coastal area and second largest brackish water area in the country. Navsari district was purposively selected for the study as it accounts for largest shrimp farming area in the state [20]. Two blocks namely Jalalpore and Gandevi ~~having~~ highest shrimp production in the district were selected for the study. From each of the sampled block, two clusters of villages were selected based on area under shrimp culture. A total of 220 shrimp farm households were selected from the district, out of which 100 were for black tiger shrimp farms and 120 for white legged shrimps. The primary data was collected from farmers using multistage stratified simple random sampling and snowball technique and was collected by personal interview method with the help of pre-tested questionnaire especially designed for the study. On the other hand, the secondary data was collected from relevant publications and books. The questionnaire elicited information on the average farm size, stocking density, feeding rates, days of culture, crops per year, equipment's used, average size of shrimps

harvested and production input quantities and costs. The farmers were asked to provide the information for the previous production year and the response rates for the survey were 97.50%.

Analytical technique

In the study, the farms with similar management strategies were grouped and the key component analysis was carried out to classify sets of variables that contributed ~~similarly~~ to the overall variability within the data set. Similar strategies were followed by [21] and [22]. The second stage of the study consist~~ed~~ of ~~grouping the~~ observations into clusters of similar characteristics. A cluster analysis was performed to classify groups of farm observations that were ~~more~~ similar in terms of key variables than observations in the other clusters such as stocking density, feeding rate, culture days, and total production etc.

Cost of cultivation

The cost of cultivation of Carp was estimated using the cost concepts defined by Commission of Agricultural Costs and Prices (CACP) ~~and mentioned in~~ [23]. These cost concepts are explained ~~below~~:

Cost A_1 = All actual expenses in cash and kind incurred in production by the producer. The items covered in cost A1 are costs on:

I. value of Post larvae (PL)
II. value of feed
III. value of medicine and fertilizers
IV. value of energy (electricity and fuel)
V. value of hired human labor (permanent and casual)

VI. land revenue

VII. interest on working capital

VIII. communication expenses

IX. depreciation on fixed capital

X. repair and maintenance

Cost A_2 = Cost A1 + Rent paid for leased-in land

Cost B_1 = Cost A1 + Interest on value of owned capital assets (excluding land)

Cost B_2 = Cost B1 + Rental value of owned land (net of land revenue) and rent paid for leased-in land

Cost C_1 = Cost B1 + Imputed value of family labor

Cost C_2 = Cost B2 + Imputed value of family labor

Cost C_3 = Cost C2* + 10 per cent of Cost C2* to (on account of managerial functions performed by farmer)

Social aspects of shrimp farming were estimated using ~~statistical tool like percentage analysis, average and measures of central tendencies~~. For each cluster identified, complete enterprise budgeting was performed based on standard techniques of [24]. ANOVA was performed on key parameters like stocking densities, survival rates, yield, feeding rate, FCR, days of culture, number of crops per year and farm size. To arrive at different efficiency measures, analysis was carried out ~~by~~ following [25];[26]

Production Function Model

The C-D function is expressed as follows:

$$ln\ (Y) = \beta_0 + \sum \beta_1\ ln\ (X_i) + \square_i \qquad (1)$$

Where, *Y* denotes output; *Xi* denotes inputs; β_0 denotes a constant; β_1 denotes model coefficients (the elasticities of production) and □$_i$ denotes the random or systematic error.

The empirical Cobb-Douglas production function for this study is expressed as follows:

$$ln\ (Y) = \beta_0 + \beta_{Fd}\ ln\ Fd + \beta_{Sd}\ ln\ Sd + \beta_{Lb}\ ln\ Lb + \beta_{Md}\ ln\ Md + \beta_{Ag}\ ln\ Ag + \beta_{Exp}\ ln\ Exp + \square_i \qquad (2)$$

Where, Yd denotes quantity of shrimp produced (kg/ha/crop): Fd denotes quantity of feed used (kg/ha): Sd denotes post larvae of seeds stocked (Pl/m^2): Lb is the labor (man-days/ha): Md is total quantities of medicine and fertilizers used (kg/ha): Ag is the actual age of the farmer involved in shrimp farming: Exp is number of years of shrimp farming experience and □$_I$ denotes the random or systematic error.

The parameters of investigational significance include:

- Inputs significant to the production process;
- Factor elasticity of each significant input; factor Elasticity (β_i) measures the marginal change in fish yield from a change in a single input while other inputs are held constant. This would be obtained from the regression analysis;
- Elasticity of scale (□) is measured by the percentage change in output with a simultaneous percentage change of equal magnitude in all inputs. The elasticity of scale is the sum of the factor elasticities in the production function

$$\square = \sum \beta_i \quad i = 1\ldots.,n \qquad (3)$$

- Allocative efficiency (AE) was determined by calculating the ratio of marginal value product (MVP) and the marginal factor cost (MFC), i.e.

$$AE = MVP/\ MFC \qquad (4)$$

$$\text{And MVP} = \beta_i \frac{\bar{y}}{\bar{x}} P_y \qquad (5)$$

Where, MFC= Price per unit of input: β_i is regression coefficient of the ith input (i=1,2,3): $\bar{Y}$ is geometric mean of output: $\bar{x}$ is geometric mean of the ith input (i= 1, 2, 3) and Py is price of output.

- The MVP was estimated at the respective geometric mean level and MFC was taken as unit price of the factor. If MVP/MFC equal unity then resource is optimally used. A value of less than unity implies ~~over-use~~ of the resource, and of greater than unity ~~under-use~~ of the resource.

Garrett's ranking technique was used to rank the constraints reported by the ~~sample~~ farmers on different factors. The shrimp farmers were asked to assign rank to all the constraints faced by them and the outcomes of such rankings were converted into score value ~~with the help of formula~~

$$\text{Percent position} = 100\ (\text{Rij} - 0.5)\ /\text{Nj} \qquad (6)$$

where, Rij is the rank given for the ith variable by the jth respondents, and Nj is the number of variable ranked by the jth respondents.

Results

Principal Component and Cluster Analyses

Principal component analysis was performed to reduce the dimensionality of the data set and for transforming the larger data into smaller ones ~~containing~~ useful information. Seven principal components were found to account for 100 % of variability in the data which revealed the internal structure of the data and also explained the variance in Table 1. The variables included in these

seven principal components culture days, area stocked, total production, man-days of labor use, medicine (including fertilizers), total seeds stocked and amount of feed used in production of each crop. The eigenvalues of the first three PCs in the bootstrapped PCA were 2.27, 1.57 and 1.23, respectively, which explained a mean of 72.41% of the total variation in the observed sample,

Table1. Principal components and eigenvalues.

Principal Component	Eigen value	Percent Variance	Cumulative Variance
1	2.27	32.39	32.39
2	1.57	22.44	54.82
3	1.23	17.58	72.41
4	0.95	13.60	86.01
5	0.62	8.86	94.87
6	0.26	3.74	98.61
7	0.10	1.39	100.00

The farms of both the *L. Vannamei* (white legged shrimp) and *P. Monodon* (Black tiger shrimp) have been categorized as low (0-2999 kg/ha/crop), medium (3000-5999 kg/ha/crop), high (6000-8999 kg/ha/crop) and very high (above 9000 kg/ha/crop) as per the yield range as shown in Table 2. The farms falling in the low yield range had a very extensive level of farming where the water exchange to the farms were completely relied on the tidal flow and used traditional shrimp farming methods. The PL were purchased from the local hatcheries where the origin of bloodstock is unknown. The farmers were low in finance and did not want to take high risks for improving their production systems. Ponds were generally harvested according to new moon and full moon pattern. During any disease outbreak, harvesting was done quickly and ponds later chemically treated. Investors in these farms were local residents and usually the farm labor was recruited from the family members or from local communities.

Table 2. Clusters identified for economic analysis

Species	Intensity Category	Yield Range

		**(Kg/ha/crop)**
Penaeus monodon	low	0-2999
Penaeus monodon	Medium	3000-5999
Penaeus monodon	High	6000-8999
Penaeus monodon	Very high	Above 9000
Litopenaeus vannamei	low	0-2999
Litopenaeus vannamei	Medium	3000-5999
Litopenaeus vannamei	High	6000-8999
Litopenaeus vannamei	Very high	Above 9000

It was observed that in Navsari the mean pond size for *L. Vannamei* and *P. Monodon* was ~~around~~ 7937 m^2 and 4672 m^2 respectively. Most of the farmers were engaged in the culture of *L. Vannamei*, very few farmers engaged ~~themselves with~~ *P. Monodon.* The white legged shrimp was most favored by the farmers ~~for it being~~ more profitable due to its early maturation, high stocking densities, very hardy species and disease resistant, wide tolerance levels and easy acceptance to food. In addition to these advantages, the species ~~is~~ prone to most pathogenic and devastating virus of shrimp (WSSV). The farmers culturing white legged shrimp were ~~also experiencing the problems like~~ low seed survival, lower growth rate, higher FCR, black gill syndrome (lack of vitamin C), white gut and body cramping (mineral imbalance).

Production performance

The clusters earlier identified and studied revealed that the farms of *Penaeus monodon* ~~(black tiger shrimp)~~ were in the yield range of low and medium clusters while the farms of *Litopenaeus vannamei* ~~(white legged shrimp)~~ ~~formed~~ medium, high and very high yielding clusters. With the level of production, the stocking density increased substantially as shown in the table 3. Yield and feeding rate also increased as the intensity of shrimp production increased. [27] obtained similar

results where the yields and feeding intensity increased with the increasing intensification of farms in the *Litopenaeus vannamei* and *Penaeus monodon* culture in Thailand and Vietnam. Higher intensity levels were followed by lower culture days. For *Penaeus monodon*, the medium and low intensity of production were associated with 146 and 158.5 culture days respectively. Similarly for *Litopenaeus vannamei*, the production clusters of very high, high and medium culture intensities were associated with 127, 141.32 and 154.33 culture days. There was a large variation observed in the stocking densities of both the black tiger shrimp and white legged shrimp. The black tiger was stocked at the rate of 12000 PL per hectare in case of lower intensified to 14000 PL for medium intensified farms. For white legged shrimp, the stocking density varied from 34130 PL/ha for medium intensified farms to 48500 Pl/ha for very high intensity groups.

Table 3: Mean values for key production parameters by categories of intensity/yield levels

	Penaeus monodon		*Litopenaeus vannamei*		
	Low	Medium	Medium	High	V. high
Stocking density (Pl/m^2)	12.00	14.00	34.13	38.56	48.50
Feeding rate (KG/ha/crop)	4238.77	5167.82	6952.63	9282.51	13858.48
Culture days	158.50	146.00	154.33	141.32	127.00
Yield Kg/ha/crop	2563.00	3804.00	4827.25	6271.98	10115.68
FCR	1.65	1.36	1.44	1.48	1.37
Harvest weight (shrimps/kg)	29.25	32.60	46.25	51.25	42.00
Survival (%)	62.40	88.57	65.40	83.30	87.00
Crops/yr.	1.00	1.00	2.00	2.00	2.00

It is observed that harvesting weight of both the species varied considerably with white legged shrimp harvested at higher counts (shrimps/kg) than the black tiger. The possible reason being early maturation in white legged shrimp, which helps in attaining the marketable size in less culture days. The lower harvesting counts for black tiger shrimp was deliberate from the farmers point of view as the species was offered higher and better prices only at lower counts leading to extended culture days of about 160. The number of crops produced per year varied for both the species. The farmers engaged with the culture of *vannamei* were generally involved in two crops per year due

to its early maturation while for *monodon* it was only a single crop per year, as it needs better pre and post stocking management. The survival rates also varied across the clusters with higher intensity clusters having better survival rates. The FCR was found highest in lower yielding intensities for both black tiger shrimps and white legged shrimps and as the intensification of farms increased the conversion ratio tend to decrease providing better benefit cost ratio.

Costs and returns in shrimp production.

~~The main objective of the farmer is to maximize the profits and to reduce the costs, so assessing the financial profitability is of key importance.~~ Table 4 shows ~~breakup of the~~ cost incurred by different levels of intensification in .both black tiger and white legged shrimps. It was observed that in *P. monodon* the total costs incurred in low and medium levels were $8,767.18 and $10,603.06 respectively. For *L. vannamei*, the cost of cultivation of very high cluster farm category was highest ($29,516.70), followed by high farms ($21,781.23) and medium farms ($15,783.93).Feed was the major cost involved in the culture that solely accounted for around 80% of the total variable costs in low and medium intensity cluster of black tiger shrimp. Similarly, for medium, high and very high clusters it accounted for approximately 70% of the total variable costs in the production of white legged shrimps. In aquaculture, feed management is the major factor affecting the water quality and production economics [28, 29]. Correct feeding pattern is important for growth and survival that greatly influence the economic performance of shrimp culture [30]. Variable costs vary with the level of output, so the costs of feed, seed, medicine, energy and labor increased with the intensity of production In the last decade, the intensity of shrimp culture has increased leading to higher stocking densities and greater feed inputs resulting in higher FCR [31,32]. Total fixed costs per hectare per year also increased with the level of production. The total fixed costs involved the cost of pond and farm building construction, purchase of aerators, feeders,

motors, generators, vehicles and others. The additional annual investments like wear and tear (depreciation) and interest on the investment are other components of the fixed cost

Table 4. ~~Item-wise break up~~ of cost of cultivation/ha in shrimp culture (US$/Ha).

	Penaeus monodon		***Litopenaeus vannamei***		
	Low	Medium	Medium	high	V. high
Feed cost	$4940.55	$6023.42	$9024.61	$12540.61	$17621.38
Seed cost	120.79	164.66	2802.73	4341.19	5652.98
Medicine and fertilizers cost	266.44	383.90	273.93	509.74	780.82
Energy (Electricity and fuel)	843.49	1017.36	777.89	878.83	1141.81
Labor	35.11	43.10	51.05	85.44	139.35
Interest on working capital	263.77	324.38	549.53	780.12	1076.79
Communication costs	9.72	10.56	9.58	12.22	10.42
Total Variable costs	6,479.87	7,967.38	13,489.32	19,148.15	26,423.55
Depreciation on fixed capital	648.20	606.50	738.92	790.90	924.80
Repairs & Maintenance	420.32	496.03	442.25	422.55	493.42
Permanent labour	501.14	535.71	357.86	413.57	528.57
Interest on fixed capital	717.65	997.44	755.58	1006.06	1146.36
**Total**	$8,767.18	$10,603.06	$15,783.93	$21,781.23	$29,516.70

The comparative estimates of different costs incurred in shrimp culture for different levels of production intensities are given in Table 5. The table shows that total cost of production (Cost C_2) per hectare of black tiger shrimp is ~~amounted to~~ $9975.52 and $13200.15 on low and medium intensity farms~~,~~ respectively. Similarly, for white legged shrimps the amount is $17678.24, $24096.78 and $33509.42 for medium, high and very high intensity groups. The different measures of costs in shrimp culture viz. costs A_1, A_2, B_1, B_2, C_1, C_2 and C_3 are higher for high intensity farms in both *P. monodon and L. vannamei*. Cost C_3 includes all the possible costs and is considered as the real cost of production in a farm situation. However, rental value of owned land and managerial

costs for the farmer can be excluded in a marginal profit situation and Cost C_1 can be taken as the standard cost of production, which includes all actual expenses expressed in cash and kind (the rental value of owned capital assets (excluding land) and imputed value of family labour).

Table 5: Cost concept wise cost of production of shrimps (US$/ha)

	Penaeus monodon		***Litopenaeus vannamei***		
	Low	Medium	Medium	high	V. high
A. Cost A1	8049.54	9605.62	15028.36	20775.17	28370.35
Rent paid for leased in land	13.89	13.89	13.89	13.89	13.89
B. Cost A2	8063.43	9619.51	15042.25	20789.06	28384.24
Interest on fixed capital	717.65	997.44	755.58	1006.06	1146.36
B. COST B1	8767.19	10603.07	15783.94	21781.23	29516.71
Rental Value of land+ Rent paid for leased in land	83.33	62.50	147.08	111.11	151.39
C. COST B2	8850.52	10665.57	15931.02	21892.34	29668.10
Imputed value of family labour	1125.00	2534.58	1747.22	2204.44	3841.32
D. COST C1	9892.19	13137.65	17531.16	23985.67	33358.03
E. COST C2	9975.52	13200.15	17678.24	24096.78	33509.42
F. COST C3	10973.08	14520.17	19446.07	26506.46	36860.36

It is observed that the cost per kilogram per hectare of shrimps produced decreased with the increase in the production intensity (Table 6). For *Penaeus monodon* the cost per kilogram per hectare was US$ 4.28 for low intensity level and later reduced to US$ 3.82 for medium intensity level. Similar pattern was observed for *Litopenaeus vannamei*, the costs decreased from US$ 4.03 for medium intensity to US$ 3.64 for very high intensity of production. The production process of the shrimps followed the economies of scale by sparing in costs and by expanding the culture. Net returns per hectare increased by increasing the level of intensification. As the production increased

across the levels of intensification, more volume of output produced resulted in greater gross recipients. For *Penaeus monodon* the net returns for the lower intensity are $12280.63 and for medium intensity ~~are~~ $20345.63 ~~stating~~ the enterprise as a profitable venture. White legged shrimp matures in short duration of time and in that case the culture is done twice a year making the net returns to $29793.98, $36207.00 and $58921.98 for medium, high and very high intensities respectively. The study reveals that as the level of intensification increased across the clusters, the profitability also increased. So in order to increase the profitability the farmer should shift ~~himself~~ from one level to another level of intensification. However, the farmers need more experience, management skills and capital to shift ~~himself from one level of production to another~~. In addition to this, the farmers should also possess the risk taking ability, as the shrimp culture is more uncertain for disease outbreak and mortality. The long run profitability of the shrimp farmers in India is affected by the increasing prices of ~~the~~ medicines, the cost of hired labors and diseases like WSSV (White Spot Syndrome Virus) which are more prone to *Litopenaeus vannamei*. Land values and construction costs greatly increases the fixed costs, which ultimately hinders the profitability. It is observed that per unit cost of production is affected greatly by the yield but the yield itself is dependent upon the stocking densities, feeding rate, culture days, aeration rate and others. Therefore, yield can be different for ~~even~~ same stocking densities per hectare depending on the level and intensities of the other management practices and inputs used.

Table 6: Returns from cultivation of shrimps on sample farms per hectare (US$)

	Penaeus monodon			***Litopenaeus vannamei***			
	Low	Medium	Average	Medium	high	V. high	Average
Yield (Kgs)	2563.00	3804.00	3183.50	4827.25	6271.98	10115.68	7071.64
Price ($/Kg)	9.07	9.17	9.12	7.11	7.11	6.56	6.93
Gross Income (GI)	23253.71	34865.80	29059.76	34343.06	44609.96	66321.35	48424.79
Cost of production ($/Kg)	4.28	3.82	4.05	4.03	4.23	3.64	3.97
Net Income= GI- Cost C3	12280.63	20345.63	16313.13	14896.99	18103.50	29460.99	20820.49

**Net income*(per year)**	**12280.63**	**20345.63**	**16313.13**	**29793.98**	**36207.00**	**58921.98**	**41640.99**
Farm Business Income=GI- CostA2	7140.74	15640.66	11390.70	4272.46	3045.74	9566.76	5628.32
Family Labour income=GI-Cost B2	14403.19	24200.23	19301.71	18412.04	22717.62	36653.25	25927.64
B:C ratio	1.12	1.40	1.26	1.53	1.37	1.60	1.50

Note: *Since *Litopenaeus vannamei* mature in short duration and 2 crop are taken in a year whereas for *Penaeus monodon* only one crop is taken and hence net income from *Litopenaeus vannamei* crop has been multiplied by 2 to obtain net income per year for comparing with *Penaeus monodon*.

Factors affecting shrimp production

It is ~~of interest to the~~ economists to understand the inputs that significantly affects the production process and the inputs having higher per unit effect on total production relative to other inputs [33]. The inputs specified for the shrimp culture were feed (Fd), Seed (Sd), Labor (Lb), Medicine (Md), Age (Ag) and Experience (Exp) of the farmers. Three forms of production function namely, linear, Cobb- Douglas, and Semi log linear were ~~tried~~ to determine the factors affecting the shrimp farming. Amongst them Cobb-Douglas form of production function was found to be the best fit on both the economic and statistical criteria. The positive production coefficients of the respective inputs in a production function implies that by increasing the intensity of input use, the output can be increased significantly. On the other hand, the negative coefficients suggests that the input should be reduced [33].

The parameters of production function were estimated by step-wise method using SPSS and the results ~~so~~ obtained are presented in table 7 for both the species. The results suggested that the production of *Peneaus monodon* was significantly influenced by the feed (Fd) at 5% level of significance and experience (Exp) at 1% level of significance, implying that if the feed is increased by 10% percent, the shrimp yield will increase by 2% and if farm experience is increased by 10% the yield will increase by 3.5%, *ceteris peribus*. The model was highly significant (ANOVA gave highly significant F- statistics, with P value significant at both 1% and 5% level of significance).

The ~~adjusted~~ R^2 was 0.63, implying that 63% of variation in production of *P. monodon* is explained by explanatory variables in the model. Similarly, the production of *L. Vannamei* was influenced by seed (Sd) and experience (Exp) at 1% level of significance and labor (Lb) at 5% level of significance. [34] and [35] reported similar findings. The ~~adjusted~~ R^2 was 0.49, implying that ~~round~~ 50% of the shrimp yield is explained by explanatory variables in the model.

Table 7. Cobb –Douglas Production Function Estimation for *Penaeus monodon* and *Litopenaeus vannamei* culture.

	Penaeus monodon		***Litopenaeus vannamei***	
	Coefficients	*Standard Error*	*Coefficients*	*Standard Error*
**Const**	6.690	3.926	0.111	2.204
**Fd**	0.199**	0.101	0.071	0.092
**Sd**	-0.245	0.278	0.384***	0.108
**Lb**	0.119	0.448	0.100**	0.052
**Md**	0.003	0.047	0.149	0.096
**Ag**	-0.021	0.162	0.065	0.127
**Exp**	0.354***	0.090	0.262***	0.063
**Mean dependent var**	7.854		8.709	
**S.D dependent var**	0.220		0.209	
**Sum squared resid**	0.360		1.015	
**S.E of regression**	0.134		0.150	
**R- squared**	0.715		0.551	
**Adjusted R-squared**	0.629		0.491	
**F value**	8.353		9.198	
**P- value (F)**	0.000129159		1.41259E-06	

indicate significance at 5% level, * indicate significance at 1% level

- Fd denotes quantity of feed used (kg/ha)
- Sd denotes post larvae of seeds stocked (Pl/m^2)
- Lb is the labor (man-days/ha)
- Md is total quantities of medicine and fertilizers used (kg/ha)
- Ag is the actual age of the farmer involved in shrimp farming
- Exp is number of years of shrimp farming experience

The models for shrimp production can be expressed as follows:

- $$Ln\ (Yd) = 6.690 + 0.199 \ln Fd - 0.245 \ln Sd + 0.119 \ln Lb + 0.003 \ln Md - 0.021 \ln Ag + 0.354 \ln Exp \quad (7)$$

- $$Ln\ (Yd) = 0.111 + 0.071 \ln Fd + 0.384 \ln Sd + 0.100 \ln Lb + 0.149 \ln Md + 0.065 \ln Ag + 0.262 \ln Exp \quad (8)$$

Equation (7) can be used to predict the *P. Monodon* production for farmers, given their production inputs, age and years of experience. Years of experience was the most powerful explanatory variable with the highest partial output elasticity of 0.354 which means a 10% increase in experience , keeping other inputs constant will increase the yield by 3.5%. Equation (8) predicts the *L vannamei* production and seed stocked was the most ~~powerful~~ variable with output elasticity of 0.384 clearly indicating 10% increase in seeds stocked would increase the output by 3.84%. The coefficients of medicine and age were not significant in both the models implying no difference in production between young and aged farmers. The level of statistical significance of the estimated production coefficient in both the models (equation 7 and 8) are encouraging and there appears to be no problem with multi-collinearity as the Variance inflation factor (VIF) values were lower than 10 (table 8)

Table 8. Variance Inflation Factor (VIF) Analysis for Multicollinearity

	Penaeus monodon	*Litopenaeus vannamei*
Variables	VIF	VIF
ln Fd	1.526	1.298
ln Sd	1.636	1.071
ln Lb	1.195	1.047
ln Md	1.250	1.073
ln Ag	1.107	1.075
ln Exp	1.824	1.362

*Note- values >10 may indicate a collinearity problem

Returns to Scale

The sum of the coefficients in the Cobb-Douglas production function (estimated elasticity function) of any production technology provides returns to scale and is of essential interest given its implications. The results of the study indicates that the *P. Monodon* production in the state has elasticity return to scale of 0.553 ($\sum\beta_i$). Since, the estimate is less than one, the production of *P. Monodon* exhibits decreasing returns to scale. This implies that a proportionate increase in inputs will lead to less proportionate increase in output. Similarly, for the *L vannamei* production the elasticity returns to scale is 0.746 ($\sum\beta_i$) also exhibiting a decreasing return to scale.

Resource use efficiency

Resource-use efficiency was estimated for those variables that had significant effect on shrimp production of both the species. It is observed that the efficiency ratio [Marginal Value Product (MVP) to Marginal Factor Cost (MFC)] for *Penaeus monodon* (black tiger shrimp) is almost equal to unity for feed indicating its economically utilized (Table 9). In the production of *Litopenaeus vannamei* (white legged shrimp), efficiency ratio is greater than unity for use of seed indicating it is underutilized and for labor the ratio is less than unity (over-utilized). Greater than unity values for efficiency ratio in seed and less than unity value for labor exhibits that the output was likely

to increase and hence revenue, if more of seed and less of labor would have been used in the shrimp production. In the previous section, the production elasticity of labour has suggested that increase in use of labour will increase shrimp production, however, this increase will not add to the profit of shrimp farmers.

Table 9. Resource-use efficiency in shrimp farming

	Feed<br>ln Fd	Seed<br>ln Sd	Labor<br>ln Lb
Penaeus monodon			
Geometric mean	12.60	-	-
Coefficients	0.20	-	-
Marginal value product (MVP)	1.13	-	-
Marginal factor cost (MFC)	1.17	-	-
Efficiency ratio (MVP:MFC)	0.97	-	-
Decision	Economically utilized	-	-
Input-use	Constant		
Litopenaeus vannamei			
Geometric mean	-	12.18	8.26
Coefficients	-	0.38	0.10
Marginal value product (MVP)	-	1.90	0.73
Marginal factor cost (MFC)	-	0.01	2.60
Efficiency ratio (MVP:MFC)	-	190.39	0.28
Decision for resource- use	-	Under utilized	Over utilized
Input –use		Increase	Decrease

Allocative efficiency of input use

To achieve the most efficient input-use, the value of the marginal value product (MVP) should be equal to its marginal factor cost (MFC) or price [36]. If the MVP of an input is greater than its

price, then the profitability can be increased by increasing the level of that input. On the other hand, if the MVP of an input is less than its price then profit can be increased by decreasing that input. In the first regression model of shrimp production (*P. monodon*) as shown in ~~t~~able 9, feed (Fd) was statistically significant and this input should be kept constant, since its MVP and MFC are almost similar. In the second regression model of shrimp production (*Litopenaeus vannamei*) in order to improve the profitability seed (Sd) should be increased since its MVP is greater than its MFC, whereas the use of labor should be decreased as its MVP is lesser than its MFC.

~~Constraints of shrimp farmers~~

Farmers were asked to rank their constraints according to their severity. Based on the response of farmers, the Garret score ~~has been~~ estimated to find the severity of each constraint and rank ~~for each constraint~~ was accorded based on Garret score and the results so obtained are presented in ~~t~~able 10. There are lot of problems which the farmers were facing so the top ten most severe problems have been discussed here. The major problem for both the *P. monodon* and *L. vannamei* was the disease problem. In addition to WSSV, the crop ~~was~~ continuously ~~suffering~~ from black gill, white gut problems, running mortality syndrome and shrimp muscle cramping. The disease management requires lot of man ~~force~~ that increased the labor cost in addition to the ~~costly~~ medicines ultimately leading to higher costs of production. The problem could be ~~solved~~ by optimum stocking densities, which prevent the overcrowding of shrimps in the ponds. Most of the farmers do not follow the stocking density protocol and overstock the ponds leading to ~~reduce the~~ dissolved oxygen and increase the stress. The second major constrain for the monodon was the high cost of inputs. One of the major input used in the shrimp production is the feed. Feed solely accounted for around 80% of the total variable costs in low and medium intensity cluster. Feed is not only the source of physiological waste but also accounts for 55% to 60% of the variables costs

in intensive and around 40% in semi intensive systems [37]. Better-feed management practice will eventually decrease the quantity of feed and ~~will~~ ultimately reduce the costs. In case of *L. Vannamei* the second constraint was availability of skilled labor. Skilled labors have specialized training and skills to perform the operation, so it positively affects the culture practice. They are very useful in taking up the complex physical and mental tasks and carry out quick decision making in any problematic situation. Some other major constraints were availability of quality seed, high rate of mortality, perishability of the produce and others.

Table 10. Constraints ~~faced by the shrimp farmers~~

Penaeus monodon			***Litopenaeus vannamei***		
**Constraints**	Garret Score	Rank	***Constraints***	Garret Score	Rank
Disease problem	96.18	1	*Disease problem*	94.67	1
High cost of input	64.51	2	*Availability of skilled labor*	74.86	2
Availability of quality seed	60.4	3	*High cost of input*	63.65	3
Availability of skilled labor	59.26	4	*Availability of quality seed*	58.48	4
High rate of mortality	51.24	5	*Perishability of produce*	52.14	5
Perishability of produce	42.38	6	*High rate of mortality*	44.02	6
Pond management	39.19	7	*Price of shrimp*	38.58	7
Timely availability	32.17	8	*Credit availability*	31.27	8
Price of shrimp	28.67	9	*Governmental schemes*	29.41	9
Governmental schemes	25.54	10	*Middle man*	29.18	10

Sustainability and intensification

Shrimp farming is one of an important productive ~~activity~~ for the population residing near coastal areas. With the increase in the level of intensification and production, the shift takes place from small farmers using their cash crop to sustain families and earn livelihoods to large farmers whose major share of production is exported and provides valuable foreign exchange. In order to increase the production the farmers are using the strategy of farm intensification that may have environmental, economic and social impacts. In the recent years, aquaculture has grown

tremendously due to growing demands (domestic and foreign markets) and promising profits leading to expansion of shrimp farms. This expansion may lead to the construction in the mangrove areas/ wetlands, which serve as valuable nursery grounds for fish and invertebrates. Intensification leads to higher stocking densities and hence the feeding rates are increased causing overfeeding and water pollution. Many bacterial and viral diseases and the cause in known to the poor management practices leading to stressful environment for shrimps mostly affect shrimp farming.

In order to make intensification successful for increasing the production in a sustainable manner, the first and foremost thing for the farmer is to possess sufficient knowledge regarding the culture practices and should have proper management skills to run the shrimp farming successfully for long run. The farmers should apply proper biosecurity measures in order to prevent intrusions of foreign pathogens for the purpose of disease prevention. The production should be technically and allocative efficient produced by better use of the available resources. The farmers should follow Good Management Practices (GMP's) in water and soil quality management, site selection and pond construction, feeding management, seed stocking and harvesting. The effluent treatment systems should be constructed and followed for assisting the farmers to improve the wastewater quality and make their farming practices more sustainable.

Discussion

The data collected from the shrimp farmers vary from each individual farmer as no two farmers have similar experience and management skills. Each farmer uses the combination of his indigenous knowledge and practice to manage his farm. The focus of categorizing levels of intensification was to channelize the farmers with similar characteristics into groups. The categories were made based on the yields of the shrimp farmers and not based on the stocking densities because stocking density may obscure the effects of intensification on cost efficiencies

and profitability. Yields also at the same time are not solely function of stocking densities but combination of aeration, feed management, medicines and fertilizers and others. [21] found that profitability of the culture could vary even with similar stocking densities. In this study the stocking densities of *P monodon* for low yielding cluster (12/m^2) was similar to medium yielding cluster (14/m^2). However, the yield was 48% higher in medium cluster than lower cluster that may be primarily due to 22% higher feeding rates in medium yielding cluster. These results provide evidences from the shrimp culture that supports the results obtained by [21] that use of multivariate tools such as cluster analysis to identify similar sets of management practices as the basis for comparative economic analyses.

The results showed that with increase in the culture investment and by more intensification of the shrimp production system, the profitability increased by short run in both the species. The level of intensification in each cluster increased the profit margins and reduced per unit costs of production by attaining the economy of scale. [38] in his study of intensification of catfish production revealed that with increase in production the profitability increases with reduction in per unit costs. [39] carried out similar study where the results obtained were opposite and per unit costs increased with the increase in the production systems. Quite often, the crop failures are blamed in post larvae quality, feed, disease outbreak, and water quality but most of the times the origin of the failure is the poor feed management [40, Piedad-Pascual 1993). The long-term profitability of the shrimp farms in India is affected by the white spot syndrome virus (WSSV) outbreaks, the problem of white gut, running mortality syndrome, the rising costs of daily labors and other macroeconomic factors like increase in land and capital costs.

The parameters of production function were estimated and it is revealed that the quantity of feed used and the level of experience significantly affect the production of black tiger shrimp. The

analysis for resource use efficiency depicted feed as an input was economically utilized and results on returns to scale revealed decreasing returns to scale. This implies that a proportionate increase in inputs will lead to less proportionate increase in output. Similarly, for the white legged shrimp the production was significantly affected by the quantity of seed, man-days of labor and experience. It was observed that amongst the significant resources seed was under-utilized and labor was over-utilized by the farmers exhibiting that the output was likely to increase and hence revenue, if more of seed and less of labor would have been used in the shrimp production, the production of white legged shrimp also exhibits decreasing returns to scale.

The study revealed that culture of white leg shrimp (*L. vannamei*) and black tiger shrimps (*P. monodon*) are both profitable in the state of Gujarat. However, the white leg shrimp culture is more economically profitable with higher productions mainly due to its early maturation leading to two culture crops per year. Majority of the farmers in the state were involved in white leg shrimp culture due to its high demands and good returns. The farmers stated the major constraint in culturing the black tiger was the extended culture period and its slow growth. There are also a large number of hatcheries producing white leg seeds and limited hatcheries producing the tiger shrimp seed. The white legged shrimp farmers produced good marketable surplus and were economically efficient. In spite of good profitability, the major problem for the farmers was disease outbreak. The diseases like running mortality, muscle cramping, and black gill were more common. The disease outbreak is possibly due to poor feed management. Improvements in feed management will reduce the dependence on fishmeal and will eventually lead to the decrease of nutrient load and reduced requirement for aeration or water exchange in the culture waters.

There are many challenges to be faced on daily basis in a shrimp culture and the farm manager should be skilled enough to manage the more intensive farms. Although the study revealed that in

both the species culture, increasing intensification of farms increases the profitability but practically shifting from one level of intensification to another level demands more capital, skills and risk taking ability. There are many case studies in Gujarat where the farmers took up the enterprise without prior knowledge and skills and invested a huge capital in the venture resulting in crop failures and ultimately losses.

Conclusion

Both shrimp practices are economically profitable while white legged shrimp farming was earned more due to its two culture crops in a year and more production compared to black tiger shrimp. It is observed that with increasing intensification of production in both the species the profitability ~~has~~ improved resulting in greater yields. With increased intensification, the costs per metric tons of shrimp produced also declined gradually. From the analysis, the main factor influencing the production of black tiger shrimp was feed and for white legged shrimp was seed and labor. The feed as input was considered efficiently used in the production process; however, the profitability of white legged shrimp would have increased if more of seed and less of labor would have been used in the ~~shrimp~~ production.

The major constraint ~~for~~ shrimp farming was the disease problem that can be ~~solved~~ by optimum stocking densities and proper feed management. The farmers and farm manager's needs to be skilled enough to understand the daily nutrient requirement as per the biomass so that the desire of attaining maximum growth does not lead to overfeed and low FCR. If the profitability is to continue ~~for~~ the long run the economic efficiency ~~as well as the~~ sustainability is to be improved. By employing technically qualified managers on farms can improve the techno-economic efficiency ~~of the~~ shrimp farming. It is recommended ~~that government through~~ extension officers

should train more farmers by imparting technology and training wherever necessary and help promote shrimp culture in the area,

Acknowledgement

The first author of this article would like to thank Chinese Scholarship Council (CSC) for the support of my doctoral degree.

References

1. National Fisheries Development Board (NFDB), 2020 March 15 Available from http://nfdb.gov.in/about-indian-fisheries
2. Global Aquaculture Alliance, India's shrimp industry adapts to COVID-19 restrictions .2020 April 5. Available from https://www.aquaculturealliance.org/advocate/indias-shrimp-industry-adapts-to-covid-19-restrictions/
3. Eanadu. Shrimp Aquaculture as an Industry in Andhra Pradesh., West Godawari District Edition, 27 March. 2020: 2
4. Economic times, 2020 April 10. Available from https://economictimes.indiatimes.com/markets/commodities
5. SeafoodSource, 2020 February 5. Available from https://www.seafoodsource.com/news/supply-trade.
6. Nakamura R. Aquaculture development in India: a model. BioScience, 1985; 35(2): 96-100.

7. Bush, S. R., P. A. M. van Zwieten, L. Visser, H. Van Dijk, R. Bosma, W. F. De Boer, and M. Verdegem. Scenarios for resilient shrimp aquaculture in tropical coastal areas. Ecology and Society 2010; 15(2):15 Available from **doi**.org/10.5751/ES-03331-150215

8. Muluk C. Baily C. Social and environmental impacts of coastal aquaculture in Indonesia. Aquaculture Development. Westview Press, Boulder, CO, 1996; 193–210. Available from **doi**.org/10.1111/j.10.4324/9780429046773-11

9. Vandergeest P, M Flaherty, and P Miller. A political ecology of shrimp aquaculture in Thailand. Rural Sociology, 1999; 64(4):573–596. Available from **doi**.org/10.1111/j.1549-0831.1999.tb00379.x

10. Thongrak S. Determinants of technical efficiency in intensive shrimp farming. Songklanakarin Journal of Science and Technology, 1995; 17:81–87.

11. Patmasiriwat D. Environmentally sensitive sector: a case study of shrimp aquaculture in Thailand based on farm survey. Thailand Development Research Institute Draft Paper, Bangkok, Thailand 1997.

12. Shang Y. C, PS. Leung, and B.H Ling. 'Comparative Economics of Shrimp Farming in Asia', Aquaculture, 1998; 164:183 – 200. Available from doi.org/10.1016/S0044-8486(98)00186-0

13. Usharani G., Chandra Reddy T, and Ravindranath. 'Economics of Brackish Water Prawn Farming in Nellore District of Andhra Pradesh', Indian Journal of Aquaculture, 1993; 8: 220 -230

14. Viswakumar M. 'Technical and Economic Consultations for Shrimp Culture in Andhra Pradesh', Fishing Chimes, 1992; 6: 30–40.

15. Jayaraman R K, Karl Marx, and V Sunderraj. ‘Economics of Improved Extensive Shrimp Farming in Vedarnyam’, In Proceedings of National Seminar on Aquaculture for 2000 A. D, Madurai Kamaraj University, Madurai, India, 1994

16. Allan GL, Maguire GB. Effects of stocking density on production of Penaeus monodon Fabricius in model farming ponds. Aquaculture, 1992; 107(1):49–66. Available from doi:10.1016/ 0044-8486(92)90049-Q

17. Arnold SJ, Sellars MJ, Crocos PJ, and Coman GJ. Intensive production of juvenile tiger shrimp Penaeus monodon: An evaluation of stocking density and artificial substrates. Aquaculture, 2006; 261(3):890–896. Available from doi:10.1016/j. aquaculture.2006.07.036

18. Krishna PV, Bhanuprakash K, Kumar Hemanth V, and Prabhavathi K. Growth, survival and production of Pacific White Shrimp (Litopenaeus vannamei) at different stocking densities under semi intensive culture systems in Andhra Pradesh. International Journal of Advanced Research. 2015; 3(9):446–452.

19. Csavas, I. Development of shrimp farming with special reference to Southeast Asia. Presented at INDAQUA ’95: Exposition of Indian Aquaculture, 27–30 January 1995, Madras, India.

20. Gujarat Fisheries Statistic, Status of shrimp production in Gujarat, Government of Gujarat, 2012; 32-36.

21. Johnson, K., C. Engle, and B. Wagner. Comparative economics of U.S. catfish production strategies: evidence from a cross-sectional survey. Journal of the World Aquaculture Society. 2014; 45(3):279–289. Available on doi.org/10.1111/jwas.12117

22. Kumar, G. and C. Engle. Economics of intensively aerated catfish ponds. Journal of the World Aquaculture Society. 2017; 48(2):320–332. Available on doi.org/10.1111/jwas.12385

23. Acharya, S.S. and Agarwal, N.N. Agricultural Marketing in India. Oxford IBH Publishing Co., New Delhi. 2002

24. Engle, C. R. Aquaculture economics and financing: management and analysis. John Wiley & Sons. 2010

25. Salim, S. S. and Biradar, R. S. Practical manual on Fisheries project formulation and management. CIFE. 2001; 26-28.

26. Raju , V.T. and Rao, D.V.S. Power function, Farm Income and profit Efficiency Measures, Economics of Farm Production and management, (Ed.) oxford and IBH Publishing co, Pvt. Ltd., New Delhi, 1993; 178-189.

27. Engle, Carole R., Aaron McNevin, Phoebe Racine, Claude E. Boyd, Duangchai Paungkaew, Rawee Viriyatum, Huynh Quoc Tinh, and Hang Ngo Minh. "Economics of sustainable intensification of aquaculture: evidence from shrimp farms in Vietnam and Thailand." Journal of the World Aquaculture society 2017; 227-239.

28. Boyd, C. E., and Tucker, C. S. Pond Aquaculture Water Quality Management Kluwer Academic Publishers. Boston, MA, 700. 1998

29. Jory, D. D. Feed management practices for a healthy pond environment in C.L. Browdy and J. S. Hopkins, editors. Swimming through troubled water, Proceedings of the special session on shrimp farming, Aquaculture ’95. World Aquaculture Society, Baton Rouge, Louisiana, USA. 1995;118-143.

30. Wyban, J. A., G. D. Pruder and K. M. Leber. Paddlewheel effects on shrimp growth, production and crop value in commercial earthen ponds. Journal of the world Aquaculture Society. 1989; 20:18-23

31. Peterson, E. L. Benthic shear stress and sediment quality. Aquacultural Engineering, 1999; 21: 85-111.

32. Peterson, E. L. and M. B. Walker. Effect of speed on Taiwanese paddlewheel aeration. Aquacultural Engineering 2002; 26:129-148

33. Kurbis, G. An Economic Analysis of Tilapia Production by Small Scale Farmers in Rural Honduras. M.Sc.Thesis. Department of Agricultural Economics and Farm Management. University of Manitoba. Winnipeg, Manitoba, 2000; 102.

34. Inoni, O.E. Allocative efficiency in pond fish production in Delta State, Nigeria: A production function approach. Agricultura Tropica Et Subtropica, 2007; 40(4):127–134.

35. Goswami, M., R.S. Biradar, and R. Sathiadhas. Techno-economic viability of rice-fish culture in Assam. Aquaculture Economics and Management, 2004; 8(5/6): 309–317.

36. Wattanutchariya, S. & T. Panayotou. The economics of aquaculture: the case of catfish in Thailand. In: Aquaculture Economics Research in Asia. International Development Research Center, Ottawa, Canada. 1982; 26–34

37. Chanratchakool P, Limsuwan C, and Turnbull J F. Health management in shrimp ponds. Aquatic Animal Health Research Institute. 1994

38. Kumar, G., C. Engle, and C. S. Tucker. Costs and risk of catfish split-pond systems. Journal of the World Aquaculture Society. 2016; 47(3):327–340. Available on doi.org/10.1111/jwas.12271

39. Goode, T., M. Hammig, and D. Brune. Profitability comparison of the partitioned aquaculture system with traditional catfish farms. Aquaculture Economics and Management. 2002; 6(1/2):19–38. Available on doi.org/10.1080/13657300209380301

40. Cruz, P. S. Shrimp feed Management: Principles and Practices. Kabukiran Enterprises, Inc. Davao City, Philippines. 1991

41. Piedad-Pascual, F. Aquafeeds and feeding strategies in the Philippines. M. B. New, A.G.J. Tacon, and I Csavas, editors. Farm-made aquafeeds. Proceeding of the FAO/AADCP, Bangkok, Thailand. 1993; 317-343

Figure 1 Click here to access/download;Figure;Fig 1.tif

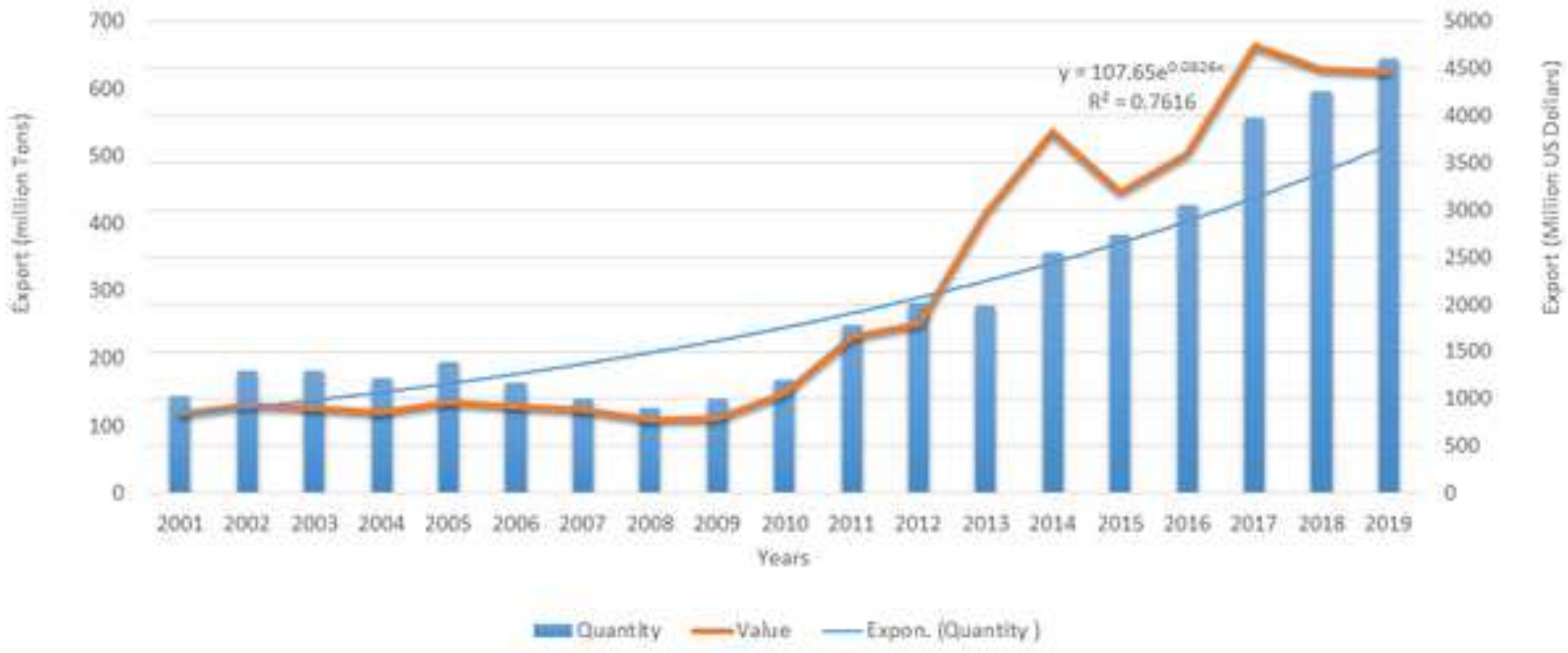

Figure 2

Click here to access/download;Figure;Fig 2.tif

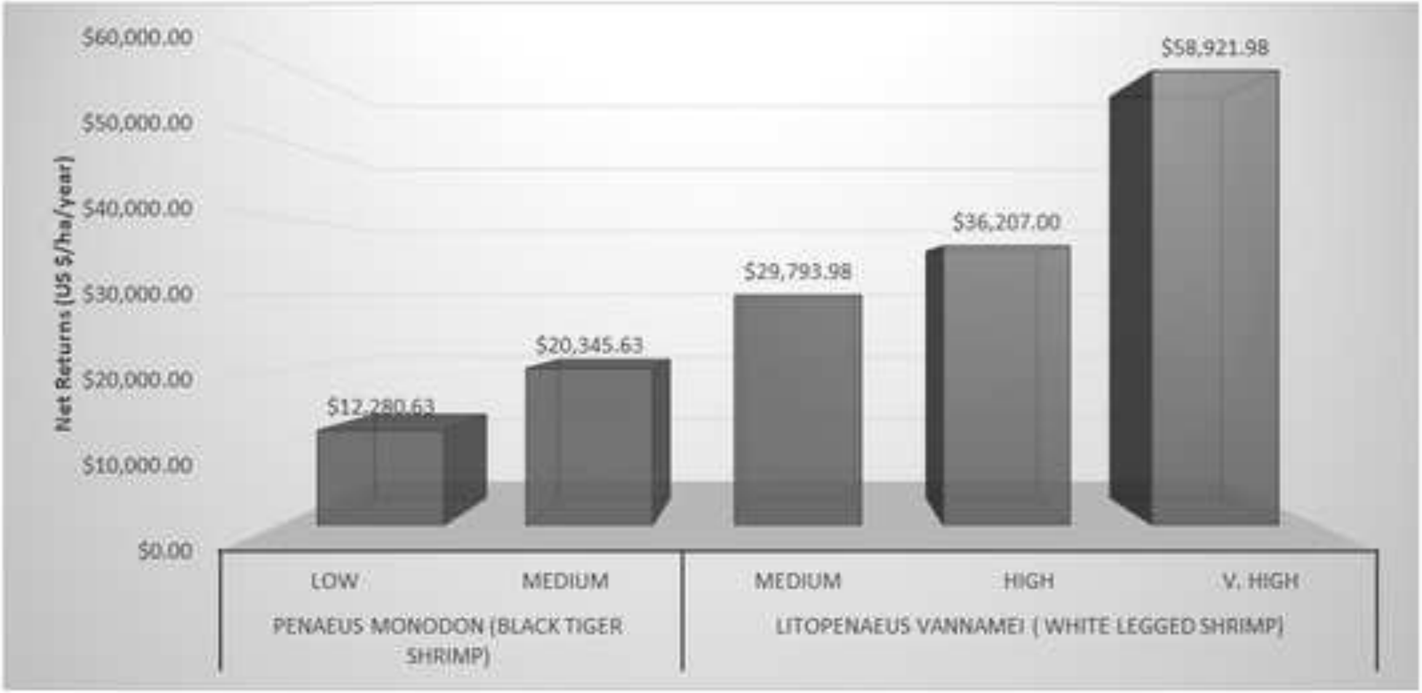

Supplement: Attachment — Submitted filename: PONE-D-20-36703 profitability reviewed.pdf [file pone.0250727.s003.pdf]
