# Supplementary material for: Comparative analysis of profitability and resource use efficiency between Penaeus monodon and Litopenaeus vannamei in India
Source: PLoS One. 2021 May 4;16(5):e0250727. doi: 10.1371/journal.pone.0250727 (PMC8096089; doi:10.1371/journal.pone.0250727)
Supplement: Attachment — Submitted filename: Response to reviewers.docx [file pone.0250727.s004.docx]

**Response to Reviewers**

Date:	Jan 11 2021 01:14PM

To:	"Yongtong Mu" ytmu@ouc.edu.cn

From:	"PLOS ONE" plosone@plos.org

Subject: PLOS ONE Decision: Revision required [PONE-D-20-36703]

Ref. No. PONE-D-20-36703

Title: Comparative Analysis of Profitability and Resource Use Efficiency for Penaeus Monodon vs Litopenaeus Vannamei in India

PLOS ONE

Dear Dr. Yongtong Mu

Thank you for submitting your manuscript to PLOS ONE. After careful consideration, we feel that it has merit but does not fully meet PLOS ONE’s publication criteria as it currently stands. Therefore, we invite you to submit a revised version of the manuscript that addresses the points raised during the review process.

Author’s response: Thank you for your interest in our study. We have made the revision according to your vital suggestions. This will significantly improve the quality of manuscript. The detailed response against each comment is described below sections.

Title: See minor amendments on Title

Author’s response: Thanks for the suggestion. Correction have been made in title as” Comparative Analysis of Profitability and Resource Use Efficiency between Penaeus Monodon and Litopenaeus Vannamei in India”

Abstract: See my inputs in text

Author’s response: Thanks for the advice. Corrections have been made.

Introduction: See my inputs in text

Author’s response: Thanks for the comments. Corrections have been made in whole MS.

Methodology: In your production function model, age and experience are not/cannot be factors of production but for productivity. Because you estimated a production function, pls expunge these variables and re-run your model, you can use the following: Pond size, Feed, seed, Medicine and or Capital inputs (depreciated). Please see other inputs in text

Author’s response: Thanks for the suggestions. Changes have been made and model has been re-run “In the new production function model variables of age and experience has been omitted and the new variable of average shrimp pond size has been added”.

Results: See my inputs in text

Thanks for the advice. Corrections have been made.

Discussion: See my inputs in text

Author’s response: Thanks for the suggestions. Changes have been made.

Conclusion: See my inputs in text

Author’s response: Thanks for the suggestions. Changes have been made.

Sincerely your’s

Yongtong Mu
